# Modulation of the gut microbiome and resistome by antibiotic use, but not by subsequent 2′-fucosyllactose intake, in adults with overweight or obesity

**DOI:** 10.1080/19490976.2026.2746814

**Published:** 2026-10-15

**Authors:** Lars M. M. Vliex, Iúri F. Godinho Mimoso, Giang N. Le, Ellen E. Blaak, John Penders

**Affiliations:** a Department of Human Biology, NUTRIM Institute of Nutrition and Translational Research in Metabolism, Maastricht University Medical Center+, Maastricht, The Netherlands; b Department of Medical Microbiology, Infectious Diseases and Infection Prevention, NUTRIM Institute of Nutrition and Translational Research in Metabolism, Maastricht University Medical Center+, Maastricht, The Netherlands

**Keywords:** Gut microbiome, resistome, antibiotics, prebiotics, body weight

## Abstract

Antibiotics are widely used to treat bacterial infections, yet overuse and misuse causes disruption of the gut microbiome and spread of antibiotic-resistant bacteria. Presence of antibiotic resistance genes (ARGs) could be a determinant of the response to antibiotics. Supplementation with prebiotics may limit the detrimental antibiotic-induced effects on the microbiome, but whether this results in changes in the gut resistome is unclear. This study investigated the impact of eight-week 2′-fucosyllactose (2′-FL) supplementation after seven-day vancomycin use in 18 adults with overweight/obesity, a subset from a double-blind placebo-controlled randomized intervention. Whole-metagenome shotgun sequencing was used to characterize the gut microbiome and resistome at baseline, after vancomycin use and after supplementation. Seven-day vancomycin use disrupted microbial richness and diversity (*p* < 0.001) and changed microbial composition. No improvements in microbial recovery were detected after eight week 2′-FL supplementation compared to placebo. Resistome richness and diversity were not affected by vancomycin use, although the presence of specific ARGs changed. No differential impact of 2′-FL supplementation on resistome richness, diversity and composition was detected. While vancomycin use disrupted the gut microbiome and affected the resistome, the detectable effects of 2′-FL supplementation were limited. Future research should investigate antibiotic- and prebiotic-induced changes in the gut resistome in more detail.

## Introduction

The worldwide consumption of antibiotics has risen in the past decades, with an estimated increase of 46% (40.2 billion defined daily doses (DDD)) from 2000 to 2018.^1^ While antibiotics are generally effective in treating bacterial infections, their overuse and misuse comes with downsides. Studies have shown the disruptive effect of antibiotic use on gut microbial diversity and composition, and these perturbations could be long lasting.^2^ ^,^ ^3^ While microbiota composition does largely recover after antibiotic use, specific taxa may remain permanently lost with potential detrimental consequences for host metabolic health.^4^ Moreover, antibiotic use in both human medicine as well as agriculture is the main driver of the emergence and spread of antimicrobial resistance (AMR).^5^ This has resulted in a global public health threat with an estimated 1.27 million deaths worldwide in 2019 alone directly attributable to AMR.^6^

In humans, the consumption of oral antibiotics has been shown to significantly impact the gut resistome, the collection of antibiotic resistance genes (ARGs) in the human microbiome.^7^ While several studies have consistently shown an increased ARG abundance and diversity upon antibiotic use and a concomitant disruption of microbial composition on a population level, these studies also highlighted that the extent of antibiotic-induced perturbations in the gut microbiome and resistome are highly individual-specific.^8^ ^,^ ^9^ ARG carriage is one important factor determining the response to antibiotic use, as it ensures survival of AMR bacteria, while susceptible bacteria perish.^10^ Besides affecting individual-specific microbial perturbations and related changes in microbial functionality upon antibiotic use, ARGs may also influence the effects of antibiotics on host metabolism. In a randomized controlled trial among males with overweight or obesity and impaired glucose metabolism, we previously showed that fecal carriage of the *vanB* resistance gene differentially impacted adipose tissue insulin sensitivity and gene expression upon oral vancomycin use.^11^

There is thus a pressing need to mitigate the negative impact of antibiotic use on the gut microbiome and its resistome. A potential strategy to improve the recovery of the gut microbiota after antibiotic disruption is through supplementation with prebiotic compounds.^12^ Prebiotics are substrates that are utilized by host microorganisms, thereby conferring a health benefit.^13^ However, limited evidence exists on how prebiotics might affect the gut resistome. One study showed that resistome richness and diversity decreased in children with obesity following a diet including prebiotics and whole-grain products for at least 30 d.^14^

We have previously shown that supplementation of 2′-fucosyllactose (2′-FL) after oral vancomycin use led to transient improvements in gut microbial resilience in adults with overweight or obesity and no impaired fasting glucose or impaired glucose tolerance.^15^ As mentioned above, the gut resistome could be of interest as a modulator of this intervention response. In these analyses, we aimed to characterize the impact of seven-day oral vancomycin use on the gut microbiome and resistome of adults with overweight or obesity using whole metagenome shotgun sequencing, and to investigate how recovery from antibiotic-induced perturbations could be influenced by 2′-FL supplementation.

## Methods

### Study design and inclusion criteria

18 participants from the total study population were selected at random for these analyses, while ensuring equal division across groups receiving 2′-FL and placebo. The methods of the original randomized double-blind placebo-controlled intervention study are presented in detail elsewhere.^15^ In brief, adults with overweight or obesity (body mass index (BMI): 25–40 kg/m^2^) and no impaired fasting glucose or impaired glucose tolerance (fasting glucose < 6.1 mmol/L and 2-h glucose < 7.8 mmol/L) were included in this study (www.clinicaltrials.gov, NCT04561284). The participants were weight stable (change in body weight < 3 kg) for at least three months before starting the study. Other exclusion criteria included suffering from chronic disease and the use of antibiotics, prebiotics or probiotics in the past three months. Participants were recruited from the area around Maastricht, The Netherlands. All volunteers gave written informed consent for participation in this study. The protocol was approved by the Medical Ethical Committee of Maastricht University Medical Center + (ref: NL73140.068.20/METC20-025). All procedures were performed according to the Declaration of Helsinki (October 2013).

**Figure 1.**
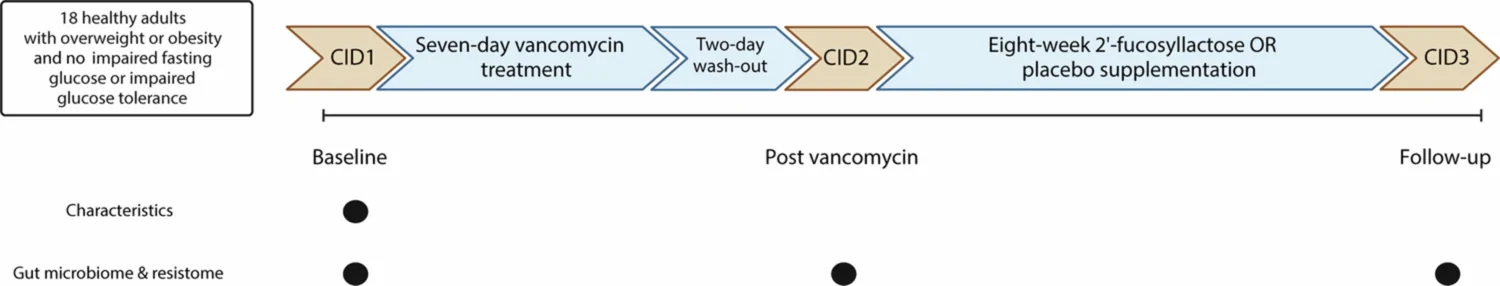
Study overview. All participants received vancomycin treatment for seven days, after which half of the participants received 2′-FL supplementation for eight weeks, while the other half received placebo. Fecal samples were collected one day prior to the clinical investigation days (CID) and handed in on these days. Created with BioRender.com.

All participants received oral vancomycin treatment (3 * 500 mg/d) for seven days. After a two-day wash-out, participants were randomized by minimization to either oral Biotis® 2′-FL supplementation (FrieslandCampina) (3 * 4 g/d) or placebo (Maltodextrin Glucidex IT 12 (Roquette)) (3 * 2 g/d) for eight weeks (Figure 1). Minimization was done using age, sex and BMI as factors. Compliance with the study protocol was assessed by counting returned vancomycin capsules and empty 2′-FL/placebo sachets. The participants and investigators were blinded to the content of the sachets. The participants were asked to maintain their habitual diet and physical activity pattern throughout the entire study. Clinical investigation days (CIDs) took place at baseline, after antibiotic use and wash-out, and after eight-week supplementation (Figure 1).

### Fecal sampling

Participants collected a fecal sample the day before each CID. They were instructed to store fecal samples in the freezer (−18 °C) and to transport them to the university maintaining the cold chain. The samples were stored at −80 °C until further analysis.

### DNA isolation

Extraction of bacterial DNA was performed using the QIAamp DNA Mini Kit (Qiagen). 150 mg of frozen feces were homogenized with 1 ml of ASL (stool lysis) buffer in a tube containing 0.5 g sterile zirconia beads (∅ 0.1 mm zirconia) (BioSpec). The cells were mechanically lysed using the FastPrep™ instrument. The supernatant was collected after centrifugation, while the pellet was mixed with 300 μl of ASL, and the above procedure was repeated. The resulting supernatants were pooled, and 260 μl of 10 M ammonium acetate was added to each lysate tube. After mixing, incubation on ice, and centrifuging, resulting supernatants were collected, one volume isopropanol was added, tubes were mixed, and the samples were incubated on ice for 60 min. After centrifugation for 15 min, supernatants were aspirated, and pellets were washed and dried. The nucleic acid pellet was resuspended in 100 μl Tris-EDTA buffer. The samples were incubated for 15 min at 37 °C with 2 μl of DNase-free RNase (1 mg/ml) and subsequently incubated with 15 μl of proteinase K and 200 μl of AL buffer for 10 min at 65 °C. 200 μl of 100% EtOH was added to the lysate and briefly vortexed. Samples were transferred to QIAamp® spin columns, washed, and eluted in 200 μl of AE buffer according to the manufacturer's protocol. Quality control of the samples was done using a 1% agarose gel. DNA concentration and purity were quantified using the Qubit 4™ fluorometer (Life Technologies) and the Nanodrop (Thermo Fisher Scientific).

### Oxford nanopore whole metagenome shotgun sequencing

Isolated DNA was repaired using NEBNext FFPE DNA Repair Mix (New England Biolabs). 50–100 ng DNA was incubated in NEBNext, FFPE DNA Repair Buffer with NEBNext, FFPE DNA Repair Mix reagents for 20 min at 20 °C. DNA clean-up was performed using AMPure XP beads. The purified DNA was eluted in 0.1× Tris-EDTA buffer.

The sequencing library was prepared using the Rapid PCR Barcoding Kit (SQK-RPB004; Oxford Nanopore Technologies). In short, per sample, 1–5 ng (3 μl) of FFPE repaired template DNA and 1 μl fragmentation mix (FRM) were gently mixed and incubated for 1 min. at 30 °C and 1 min. at 80 °C. Tagmentation was performed by adding 20 μl nuclease–free water, 1 μl pre-determined barcode (1–12A) and 25 μl 2× Q5 HotStart master mix (New England Biolabs). Amplification was done using following conditions: an initial denaturation for 3 min. at 95 °C, followed by 14 cycles of 15 s 95 °C, 15 s 56 °C and 6 min 65 °C, and a final extension for 6 min at 65 °C. AMPure beads were used for clean-up and elution was done in 10 mM Tris-HCL (pH 8) 50 mM NaCl. DNA concentration was measured using the Qubit 4 fluorometer. Approximately 200 ng library was loaded onto an R9.4.1 flowcell, ultra-high accuracy basecalling was conducted using the MinKnow software (version 20.06.2, Oxford Nanopore Technologies), and the run was ended when all barcodes had minimal 500 Mbp of basecalled sequences (48–72 h).

### Bioinformatics and statistics

Adapter sequences were removed from the reads using Porechop (version 0.2.4). Newly trimmed reads were mapped using minimap2^16^ to the reference genome with accession GCA_000001405.15_GRCh38. SAMtools was used to remove the human contamination reads and to convert the alignment file to FASTQ.^17^ Kraken2 was deployed with the confidence setting of 0.1 for taxonomic identification.^18^ Bracken was used to identify the relative abundance of detected species.^19^ Metagenomic reads were assembled using Flye (version 2.8.2).^20^ ARGs were identified using ABRicate v1.0.1 against the NCBI AMRFinderPlus nucleotide database (version 3.10/2021-03-01.1, downloaded March 2021), using default thresholds of ≥80% nucleotide identity and ≥80% coverage.^21^ Read quality summary statistics were calculated with seqkit v2.12.0.

Statistical analysis and data visualization were done using R (version 4.2.3). Graphs were made using the microViz (version 0.11.0—Copyright © 2022 David Barnett),^22^ ggplot2 (version 3.4.1) and ggstatsplot (version 0.11.0)^23^ R packages. Analyses of α- and β-diversity were performed using the vegan (version 2.6.4) and phyloseq (version 1.42.0) packages.^24^ ^,^ ^25^

Data are expressed as mean ± standard error of the mean (SEM) where possible, with a significance level of *p* < 0.05 (or *q* < 0.05, corrected for false discovery rate (FDR) using either Benjamini–Hochberg or Holm's methods where applicable). Differences in participant characteristics between baseline and post vancomycin use were assessed using paired *t*-tests. Differences between treatment groups were analyzed using independent *t*-tests. Linear mixed-effect models were used to assess changes in participant characteristics during eight-week 2′-FL supplementation compared to placebo, using the lme4 and lmeTest packages.^26^ ^,^ ^27^ Treatment and time were included as fixed effects, and participant IDs as random effects. Change in characteristics during seven-day vancomycin use and BMI at baseline were used as covariates unless stated otherwise.

Changes in microbial and resistome α-diversity (Chao1 estimated richness and Shannon diversity) over time were compared between 2′-FL and placebo using linear mixed-effect models, with the same fixed and random effects as described above. Change in α-diversity metric during seven-day vancomycin use and BMI at baseline were used as covariates. In case of a significant time*treatment interaction effect, post-hoc testing was done to determine differences between 2′-FL and the placebo. Analysis of variance (ANOVA) with a paired *t*-test with Holm's correction was performed to compare changes in α-diversity indices over-time within the treatment groups. Principal component analysis was performed on centered-log ratio transformed species counts, as well as on ARG count data. Jaccard distance was used to determine sample dissimilarity between and within individuals.

For downstream statistical analysis, raw bacterial species and ARG counts were filtered (eliminating species detected in <20% of samples and ARGs detected in <10% of samples) to reduce the number of comparisons. Changes in the relative abundance of bacterial species over-time were analyzed using Wilcoxon signed-rank test. Changes in ARG presence were analyzed using McNemar's test with continuity correction. Correlations between bacterial species and ARGs were assessed using Spearman's correlation.

## Results

To study the effects of vancomycin use and subsequent 2′-FL supplementation on the gut microbiome and resistome, 18 adults with overweight or obesity received a seven-day vancomycin treatment. Participants were then randomly assigned to either 2′-FL or placebo supplementation for eight weeks. The study population had a mean age of 45 ± 3 y and a mean BMI of 32.2 ± 0.9 kg/m^2^ (Table 1). There were no differences in baseline characteristics between the groups that would receive 2′-FL and placebo. Except for a decrease in systolic blood pressure in the total group (125 ± 2.2 vs. 121 ± 2.5; *p* = 0.021), these characteristics did not change during seven-day vancomycin use. There was no differential change in the characteristics during eight-week 2′-FL supplementation compared to placebo.

**Table 1.** Characteristics of the participants included in the current analyses at baseline and throughout the study period.

		Baseline			Post vancomycin			Follow-up		
	Total group (*n* = 18)	Placebo (*n* = 9)	2′-FL (*n* = 9)	*p*-value	Placebo (*n* = 9)	2′-FL (*n* = 9)	*p*-value*	Placebo (*n* = 9)	2′-FL (*n* = 9)	*p* Treatment*Time^†^
Sex (male/female)	9/9	3/6	5/4							
Age (y)	46 ± 3.4	43 ± 5.3	48 ± 4.2	0.663						
Weight (kg)	96.0 ± 4.0	92.9 ± 4.6	99.1 ± 6.5	0.291	92.7 ± 4.9	98.6 ± 6.8	0.105	92.9 ± 5.3	98.7 ± 6.8	0.838
BMI (kg/m^2^)	32.6 ± 1.0	32.1 ± 1.3	33.1 ± 1.4	0.240	32.0 ± 1.4	32.9 ± 1.5	0.098	32.1 ± 1.4	33.0 ± 1.5	0.987
Waist (cm)	104.6 ± 2.6	99.9 ± 2.8	108.8 ± 3.7	0.100	99.2 ± 2.7	107.7 ± 4.0	0.198	98.7 ± 3.4	108.5 ± 4.2	0.484
Hip (cm)	109.2 ± 2.1	106.3 ± 2.7	111.7 ± 3.0	0.239	107.6 ± 2.4	111.3 ± 3.6	0.655	106.1 ± 3.3	110.1 ± 3.2	0.931
W/H ratio	0.96 ± 0.01	0.94 ± 0.02	0.97 ± 0.01	0.181	0.92 ± 0.03	0.97 ± 0.02	0.253	0.93 ± 0.02	0.98 ± 0.02	0.625
SBP (mmHg)	125 ± 2.2	123 ± 2.1	127 ± 3.6	0.410	121 ± 3.4	121 ± 3.8	0.021	124 ± 2.6	125 ± 4.3	0.476
DBP (mmHg)	84 ± 1.6	84.5 ± 1.4	83.4 ± 2.9	0.744	84 ± 3.0	81 ± 2.9	0.298	83 ± 3.2	83 ± 2.3	0.874
HR (bpm)	72 ± 2.9	76.7 ± 4.0	66.8 ± 3.0	0.081	76 ± 5.2	70 ± 3.2	0.256	72 ± 3.7	69 ± 2.8	0.247
Fasted glucose (mmol/L)	5.3 ± 0.1	5.3 ± 0.2	5.4 ± 0.1	0.878	4.9 ± 0.2	5.1 ± 0.2	0.902	4.9 ± 0.1	5.2 ± 0.2	0.596
2-h glucose (mmol/L)	5.2 ± 0.3	5.0 ± 0.4	5.4 ± 0.4	0.360	5.6 ± 0.5	6.2 ± 0.5	0.125	5.8 ± 0.6	6.2 ± 0.6	0.699
HbA1c (mmol/mol Hb)	35.7 ± 0.7	35.7 ± 0.8	35.7 ± 1.1	0.823						

^*^ : denotes *p*-value of paired *t*-test comparing baseline and post vancomycin (whole group).

^†^ : denotes *p*-value of the Treatment*Time interaction from the linear mixed effectmodel comparing 2′-FL and Placebo from post vancomycin to follow-up. For the linear mixed of weight, BMI, waist and hip, BMI at baseline was not included as covariate.

Abbreviations: 2′-FL: 2′-fucosyllactose; BMI: body mass index; W/H ratio: waist-to-hip ratio; SBP: systolic blood pressure; DBP: diastolic blood pressure; HR: heart rate; HbA1c: glycated hemoglobin.

### Vancomycin use and 2′-FL supplementation modulate the gut microbiome

Oxford nanopore whole metagenome shotgun sequencing was used to characterize the changes in the gut microbiome and resistome after seven-day vancomycin use and subsequent eight-week 2′-FL or placebo supplementation. After trimming and host removal, a median of 355859 reads per sample (IQR: 288021–490392) were retained, corresponding to a median yield of 1.40 Gb per sample (IQR: 1.07–1.80 Gb). The median mean read length was 3752 bp (IQR: 3265–4287 bp), with a median read N50 of 4058 bp (IQR: 3477–4599 bp). The median Q20 base proportions were 64% (IQR: 61%–66%).

Seven-day vancomycin use significantly decreased Chao1 estimated species richness (86 ± 3.8 vs. 51 ± 4.5; *p* < 0.001) and Shannon diversity (2.83 ± 0.06 vs. 1.73 ± 0.09; *p* < 0.001) when comparing the whole group. A similar decrease was seen when comparing within groups that would later receive 2′-FL or placebo (Figure 2A and B). As expected, shifts were statistically significant, with no differences between the groups that would later receive 2′-FL or placebo (Supplementary Table 1). In line with the decreased microbial richness and diversity, the gut community structure drastically shifted upon vancomycin use (Figure 2C).

Microbial inter-individual dissimilarity, illustrated by Jaccard distance between samples, significantly increased after vancomycin use (0.494 ± 0.005 vs. 0.765 ± 0.006; *p* < 0.001), indicating that antibiotic use caused samples of different individuals to become less similar. These changes during vancomycin use were comparable and statistically significant, with no difference between the groups that would later receive 2′-FL or placebo (Supplementary Figure 1).

Vancomycin had clear disruptive effects on gut microbial composition, as 94 out of 121 bacterial species changed significantly (*q* < 0.05) (Supplementary Table 2). The relative abundance of most species decreased during vancomycin use (80/94), while 14 species expanded. Of the 25 most abundant bacterial species across all the samples, the relative abundance of *Blautia sp. SC05B48, Ruminococcus gnavus, Faecalibacterium prausnitzii*, *Bifidobacterium longum, Roseburia intestinalis, Ruminococcus torques, Bifidobacterium adolescentis, Anaerostipes hadrus, Coprococcus comes, Collinsella aerofaciens, Blautia obeum* and *[Clostridium] innocuum* decreased during vancomycin use (*q* < 0.05), while relative abundance of *Escherichia coli*, *Lactiplantibacillus plantarum, Lacticaseibacillus rhamnosus*, *Acidaminococcus intestinalis, Klebsiella pneumoniae, Megamonas funiformis, Lacticaseibacillus paracasei* and *Pediococcus acidilactici* increased (*q* < 0.05) (Supplementary Figure 2).

**Figure 2.**
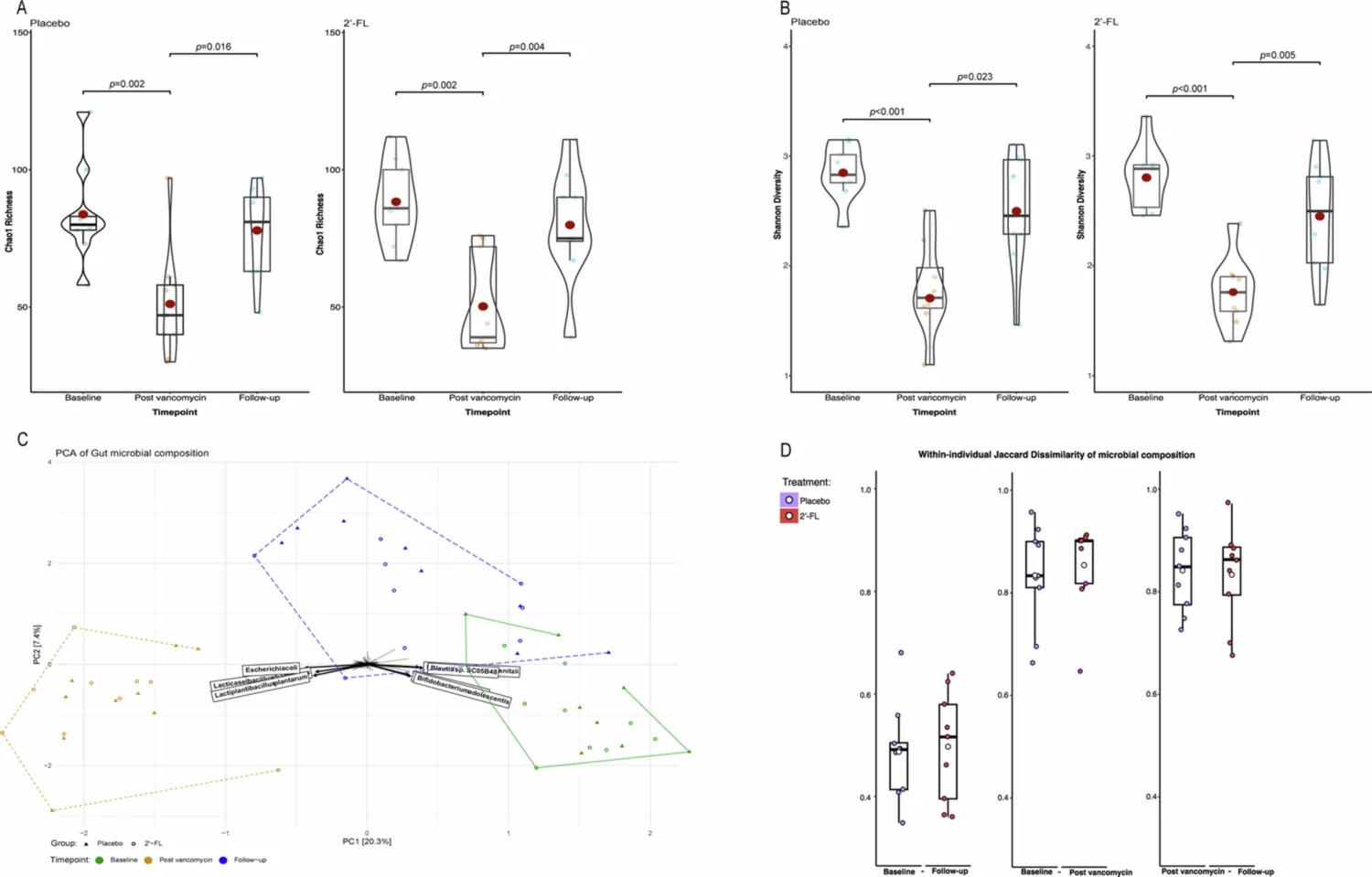
The effect of seven-day vancomycin use and eight-week 2′-FL or placebo supplementation on Chao1 estimated species richness (A), Shannon diversity (B), microbial community structure illustrated by principal component analysis ordination (centered-log ratio transformed species abundance) (C) and within-individual Jaccard distance (sample dissimilarity) (D) over-time. Sample size *n* = 9 for both the 2′-FL and the placebo group. Comparisons where made within groups over-time (A, B) and between groups (D). 2′-FL: 2′-fucosyllactose.

Next, we examined the recovery of the vancomycin-induced microbial perturbations. Both Chao1 and Shannon diversity indices increased during eight-week supplementation of 2′-FL and placebo (Figure 2A and B), although there was no detectable difference between the groups (*p*Time*Treatment = 0.901 and 0.648, respectively). A return towards the baseline community structure was seen post supplementation, although the samples did not fully cluster with the baseline samples (Figure 2C). The inter-individual Jaccard dissimilarity significantly decreased during eight-week 2′-FL or placebo supplementation compared to post-vancomycin use (0.769 ± 0.010 vs. 0.621 ± 0.011 and 0.780 ± 0.012 vs. 0.569 ± 0.012; *p* < 0.001 for both), indicating that samples of individuals in the same group became more similar (Supplementary Figure 1).

On a compositional level, eight-week 2′-FL supplementation caused significant changes in 13 bacterial species, of which 11 expanded and 2 decreased compared to post-vancomycin (*q* < 0.05) (Supplementary Table 2). Eight-week placebo supplementation caused changes in 30 bacterial species, of which 27 expanded and 3 diminished (*q* < 0.05). Of note, no species were detected to solely change in the 2′-FL group. Among the 25 species with the highest abundance across all the samples, the relative abundance of *Ruminococcus gnavus, Faecalibacterium prausnitzii*, *Roseburia intestinalis* and *Coprococcus comes* increased under eight-week 2′FL or placebo supplementation (*q* < 0.05), while *Escherichia coli*, *Lactiplantibacillus plantarum* and *[Clostridium] innocuum* decreased (Supplementary Figure 2, Supplementary Table 2)*. Blautia sp. SC05B48* and *Blautia obeum* significantly increased only under eight-week placebo supplementation (*q* < 0.05). These data indicate that the relative abundance of bacterial species returned to baseline levels under eight-week supplementation.

The within-subject Jaccard dissimilarity between the post-vancomycin and post-supplementation (CID2–CID3) was not significantly different between 2′-FL and placebo groups (0.833 ± 0.032 vs. 0.842 ± 0.027; *p* = 0.863) (Figure 2D), indicating that shifts in the microbial community structure between the end of antibiotic exposure and the end of the eight-week supplementation were comparable between the intervention groups. The within-subject dissimilarity between baseline and post-vancomycin (CID1–CID2) and post-vancomycin and post-supplementation (CID2–CID3) was higher compared to baseline and post-supplementation (CID1–CID3) in both the 2′-FL and placebo groups (Figure 2D), indicating a return towards baseline community structure after supplementation, as samples at baseline and post-supplementation were more similar to one another (CID1–CID2 vs. CID1–CID3: *p* < 0.001; CID2–CID3 vs. CID1–CID3: *p* < 0.001 (full group)).

### Vancomycin use and 2′-FL supplementation affect the fecal resistome

In contrast to the microbiome, the Chao1 estimated resistome richness and Shannon diversity did not change upon vancomycin use (Chao1: 28 ± 5.3 vs. 39 ± 10.2; *p* = 0.502, Shannon: 2.01 ± 0.18 vs. 1.87 ± 0.20; *p* = 0.572) when comparing the whole group. In line, no changes were detected when comparing within groups that would later receive 2′-FL or placebo (Figure 3A and B, Supplementary Table 1). The inter-individual Jaccard dissimilarity between resistome samples significantly increased after vancomycin use (0.775 ± 0.010 vs. 0.910 ± 0.008; *p* < 0.001), indicating that vancomycin did impact the fecal resistome and made the resistome of different individuals less similar. These findings were comparable and statistically significant, with no difference between the groups that would later receive 2′-FL or placebo (Supplementary Figure 3).

Using the assembled contigs generated from whole metagenome shotgun sequencing in ABRicate, 116 ARGs could be identified across the three clinical investigation days (Supplementary Figure 4). The presence of specific ARGs was modulated by vancomycin use: *blaEC-8* was significantly more frequently detected (*p* = 0.023), while *aadE*, *ermB*, *tet(32)*, *tet(O)* and *tet(Q)* were less frequently detected (*p =* 0.006, *p* = 0.003, *p* = 0.003, *p* < 0.001, and *p* = 0.009) (Table 2). Several genes not detected at baseline could be detected post vancomycin use: *aph(3)-Ib*, *aph(6)-Id*, *fosA_gen*, *oqxA10*, *oqxB9*, and *sul2*. Conversely, *aad9*, *catA13*, and *dfrF* could no longer be detected post vancomycin use.

**Figure 3.**
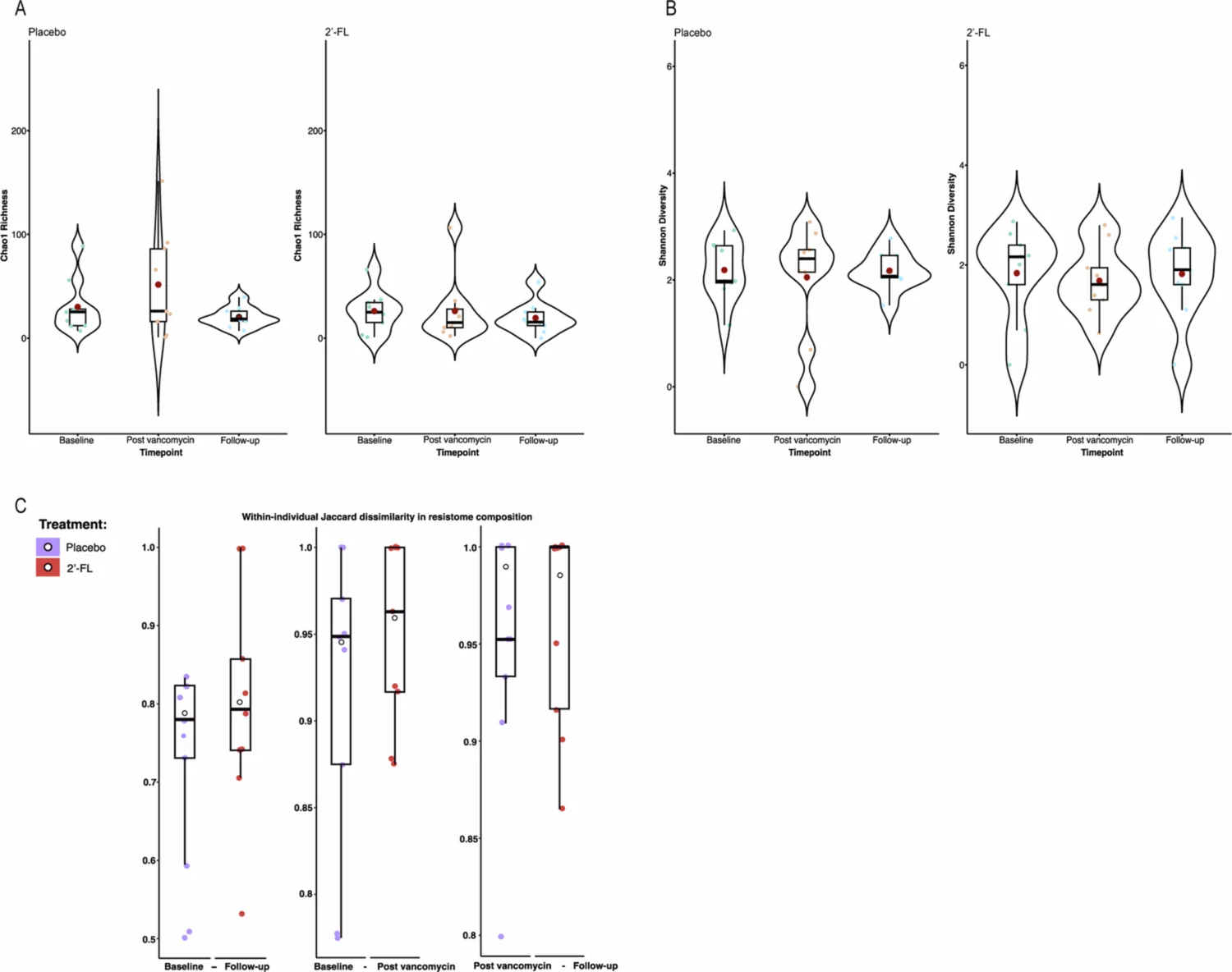
ARG estimated richness (Chao1) (A), Shannon diversity (B) and within-individual Jaccard distance (sample dissimilarity) over-time (C) based on ARG relative abundance. Sample size *n* = 9 for both the 2′-FL and the placebo group. Comparisons were made within groups over-time (A, B) and between groups (C). 2′-FL: 2′-fucosyllactose.

**Table 2.** Overview of significant changes in ARG presence throughout the study period.

					Placebo			2′-FL			
	Baseline	Post vancomycin	*p-*value		Baseline	Post vancomycin	Follow-up	Baseline	Post vancomycin	Follow-up	*p-*value
*blaEC-8*	1	8	0.023	*tet(40)*	3	1*	7*	3	0	4	0.041
*ANT(6)-Ia*	12	1	0.006	*ANT(9)-Ia*	2	0	5	3	0	4	
*ErmB*	12	1	0.003	*ANT(6)-Ia*	5	0	8	7	1	3	
*tet(32)*	12	1	0.003	*catA13*	2	0	5	3	0	3	
*tet(O)*	16	2	<0.001	*fosA_gen*	0	6	0	0	2	0	
*tet(Q)*	12	2	0.009	*oqxA10*	0	5	0	0	1	0	
											
*APH(3″)-Ib*	0	5		*tet(O)*	9	2	9	7	0	8	
*APH(6)-Id*	0	6									
*FosA*	0	8									
*oqxA10*	0	6									
*oqxB9*	0	6									
*sul2*	0	6									
*ANT(9)-Ia*	5	0									
*catA13*	5	0									
*dfrF*	10	0									

2′-FL: 2′-fucosyllactose. Filtered ARG, present in >10% of all samples. Placebo: *n* = 9; 2′-FL: *n* = 9. * Indicates the comparison that was significant.

There were no detectable significant changes in resistome Chao1 and Shannon diversity during the subsequent eight-week supplementation with 2′-FL or placebo Compared to post-vancomycin (*p*Treatment*Time = 0.172 and 0.862, respectively) (Figure 3A and B, Supplementary Table 1). In both the 2′-FL and placebo groups, the inter-individual Jaccard dissimilarity between resistome samples significantly decreased during the eight-week supplementation period Compared to post vancomycin (0.931 ± 0.014 vs. 0.858 ± 0.017 and 0.898 ± 0.019 vs. 0.757 ± 0.016; *p* = 0.003 and *p* < 0.001, respectively), indicating that samples of individuals in the same group became more similar again during supplementation (Supplementary Figure 3).

Eight-week 2′-FL supplementation did not lead to significant modulation of specific ARGs compared to post-vancomycin, except for *tet(O)*, which could not be detected post-vancomycin but was detected in 8/9 individuals post supplementation (Table 2). After eight-week placebo supplementation, *tet(40)* had expanded significantly: the gene was detected in 7 individuals post supplementation compared to 1 individual post vancomycin use (*p* = 0.041).

Furthermore, *tet(O)* was detected in all nine individuals post placebo supplementation, while the gene could only be detected in 2/9 post vancomycin. *aad9*, *aadE*, and *catA13* were not detected post vancomycin, but were detected again post placebo supplementation. Conversely, *fosA_gen* and *oqxA10* were no longer detectable post placebo supplementation, while they could be detected in several samples post vancomycin. Overall, a pattern is visible in the expansion/decrease in ARGs during the study period. Genes either expanded during vancomycin use and then diminished during the supplementation period or diminished during antibiotic use but expanded again during eight-week supplementation (Supplementary Table 3). None of the genes that significantly expanded during vancomycin use remained present in all individuals at the end of the study period.

The within-subject Jaccard dissimilarity between post-vancomycin and post-supplementation (CID2–CID3) did not differ between the 2′-FL and placebo groups (0.959 ± 0.018 vs. 0.946 ± 0.021; *p* = 0.712), indicating that eight-week supplementation with 2′-FL did not have a detectable differential effect on overall resistome composition compared to placebo (Figure 3C). The within-subject dissimilarity of resistome samples was higher between baseline and post vancomycin (CID1–CID2) and post vancomycin and post supplementation (CID2–CID3) compared to baseline and post supplementation (CID1–CID3), indicating a return towards the baseline resistome community structure, as the baseline and post-supplementation samples were more similar (CID1–CID2 vs. CID1–CID3: *p* < 0.001; CID2–CID3 vs. CID1–CID3: *p* < 0.001) (Figure 3C). These findings show that, besides changing the fecal microbiome, vancomycin had the ability to drive changes in the fecal resistome.

Finally, we analyzed correlations between bacterial species and specific ARGs in our study population. Several resistance genes were strongly positively correlated with many bacteria, and of these *tet(O)* correlated with most bacterial species: *Roseburia intestinalis* (Spearman's *ρ* = 0.75)*, [Ruminococcus] gnavus* (ρ *= *0.72), *Roseburia hominis* (ρ*=*0.72), *Clostridioides difficile* (*ρ* = 0.70) and more (Figure 4, Supplementary Table 4). Additionally, *blaEC−8* correlated with *Veilonella dispar* (*ρ* = 0.68), and *aadE* correlated with *Faecalibacterium prausnitzii* (*ρ* = 0.62).

Overall, an interesting pattern of correlations could be seen, with a clear distinction between two groups of genes: *aad9, aadE, catA13, dfrF, ermB, tet,^28^ tet(O)* and *tet(Q)* were generally positively correlated with bacterial species decreasing under vancomycin use (e.g. *Blautia sp. SC05B48, [Ruminococcus] gnavus, Faecalibacterium prausnitzii*, *Bifidobacterium longum, Roseburia intestinalis*) (Figure 4). On the other hand, *aph(3)-Ib, aph(6)-Id, blaEC−8, fosA_gen, oqxA10, oqxB9* and *sul2* were generally positively correlated with bacterial species that expanded during vancomycin use (e.g. *Escherichia coli, Lactiplantibacillus plantarum* and *Lacticaseibacillus rhamnosus*).

**Figure 4.**
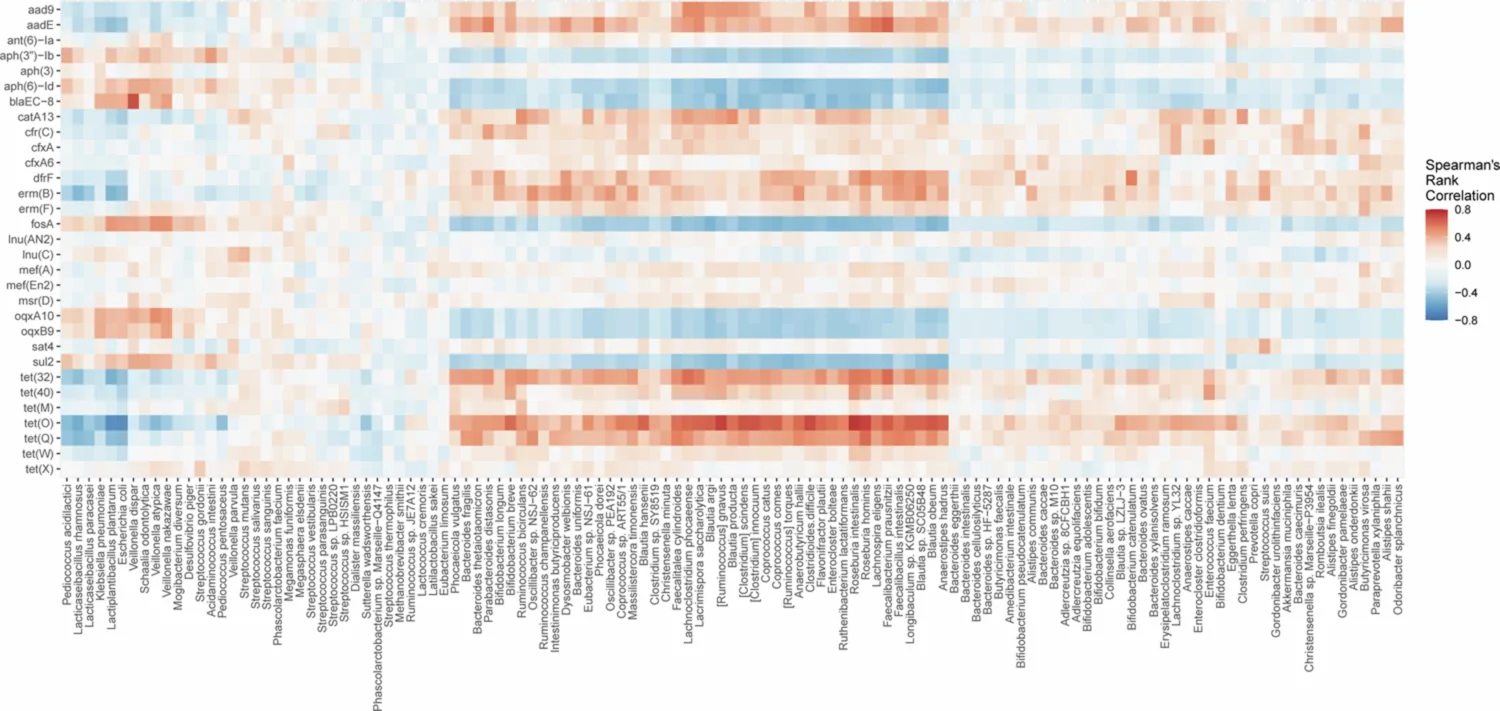
Heatmap showing correlations between ARG and bacterial species across all samples and timepoints. Red color indicates positive correlation, blue color indicates negative correlation.

## Discussion

The aim of this study was to characterize the impact of seven-day vancomycin use on the gut microbiome and resistome of adults with overweight or obesity, as well as to investigate the impact of subsequent eight-week 2′-FL supplementation. To this end, Oxford Nanopore whole metagenome shotgun sequencing was applied. A clear benefit of this metagenomic sequencing over the commonly used 16S rRNA gene sequencing approach is the measure of functional capacity that is gained by investigating the whole metagenome.^29^ This is especially relevant for studies such as ours aiming to investigate the impact of antibiotic use, as the effect of presence of antibiotic resistance genes can be studied.

Our current analyses showed that seven-day vancomycin use led to major disruptions in microbial composition, estimated richness and diversity, a finding in line with literature.^3^ ^,^ ^30^ ^,^ ^31^ Seven-day oral vancomycin use has previously been shown to cause decreased gut microbial diversity in males with obesity and metabolic syndrome as well as in males with obesity and insulin resistance.^3^ ^,^ ^30^ Furthermore, 14-d oral vancomycin use decreased microbial diversity and shifted microbial community structure compared to baseline in adults with obesity and impaired glucose tolerance.^31^ These studies showed decreased relative abundance of *Blautia obeum*, *Ruminococcus gnavus*, *Faecalibacterium prausnitzii* and *Collinsella aerofaciens* after vancomycin use, corresponding with our current findings.^3^ ^,^ ^30^ ^,^ ^31^ In further agreement with our findings, these studies showed significant expansion of *Lactiplantibacillus plantarum*, *Escherichia coli* and *Klebsiella pneumoniae* after vancomycin use. One interesting difference is the clear significant decrease in the relative abundance of *Bifidobacterium* (*B. adolescentis, B. bifidum* & *B. longum*) shown in our results, which was not mentioned in previous studies. One reason for this may be the difference in methodologies used to assess the gut microbial composition. Noteworthy, Palleja et al. (2018) showed that a four-day treatment with an oral antibiotic cocktail (vancomycin, gentamycin and meropenem) caused significant disruptions in the gut microbial composition and diversity in healthy males.^10^ As expected, the disruptions in microbial composition were more severe compared to studies only using vancomycin. The disruptions extended beyond the species named above, and this included a decrease in *B. adolescentis* and *B. longum*.

No differential effect of eight-week 2′-FL supplementation was detected Compared to placebo on microbial recovery. The bacterial species that changed under 2′-FL supplementation also changed in the placebo group, indicating no added effect of 2′-FL. Specifically: relative abundance of *Roseburia intestinalis, Coprococcus comes, Ruminococcus gnavus* and *Faecalibacterium prausnitzii* increased in both groups, while *Escherichia coli* and *Lactiplantibacillus plantarum* decreased. The species that significantly increased only in the placebo group include *Blautia sp. SC05B48* and *Blautia obeum*. These results correspond to our findings in the original study population, albeit with a different methodology, underlining the limited impact of 2′-FL supplementation on the recovery of individual bacterial taxa.^15^

As opposed to the disruptions in the gut microbiome, our results showed that resistome richness and diversity were not affected by vancomycin use. This discrepancy may be explained by the taxonomic changes elicited by vancomycin use. While a strong decrease in microbial diversity and richness was found as taxa diminished due to the effects of vancomycin, other taxa were able to expand. Among these expanding taxa were members of the *Enterobacteriaceae* family, bacteria well-known for carrying a higher amount of ARGs.^28^ ^,^ ^32^ So while microbial diversity and richness decreased, resistome richness and diversity remained stable.

Our findings are in contrast with previous studies investigating the effects of other classes of antibiotic on the gut resistome: in adults with chronic back pain, three-month amoxicillin treatment increased ARG abundance and diversity, a change that persisted at least until nine-month follow-up.^9^ Of interest, the long-term perturbations did not match the recovery of the gut microbiome in these nine months, as, with the exception of *Lachnospira pectinoschiza,* the microbial composition had recovered to baseline, as had the estimated richness. Similarly, seven-day cefprozil treatment has been shown to lead to resistome diversification in healthy adults, a change that persisted through 90-d follow-up.^33^ A persistent increase in ARG relative abundance, up to six months after treatment cessation, was furthermore shown after five-day azithromycin or cefpodoxime treatment in healthy adults.^34^ The main explanation for the difference in findings across studies may be the type of antibiotic used, as each antibiotic class has a specific mode of action. Further factors that could explain the difference in results include the treatment duration, characteristics of the study population and the baseline gut microbiome, as well as differences in the sequencing and analysis methods. This variability in studies investigating the gut microbiome after antibiotic use makes it difficult to compare results.

While there were no significant changes in resistome richness and diversity during seven-day vancomycin use, changes in the presence of specific ARGs were apparent: *blaEC-8*, a class C β-lactamase working against cephalosporins, was significantly more present after seven-day vancomycin use. Several other ARGs could be detected after vancomycin use while they were not detected at baseline: *aph(3)-Ib* and *aph(6)-Id* (coding for aminoglycoside phosphorylases), *fosA_gen* (fosfomycin thiol transferase), the *sul2* gene working against sulfonamides, as well as *oqxA10* and *oqxB9* (RND (resistance-nodulation-division) efflux pumps). These genes were positively correlated with bacteria that expand during vancomycin use, such as the opportunistic pathogen *Escherichia coli,* as well as both *Lactiplantibacillus plantarum* and *Lacticaseibacillus rhamnosus* which have intrinsic resistance against vancomycin.^35^ ^,^ ^36^ Expansion of these ARGs therefore seems to be linked to expansion of bacterial species that take advantage of the general depletion of the gut microbiome during vancomycin use. The relative abundance of these potentially resistance-carrying species decreased and recovered to baseline levels during the eight-week recovery period, and a similar pattern was seen for the ARGs.

Several ARGs were significantly less detected after vancomycin use: this was the case for *aadE* (coding for an aminoglycoside nucleotidyltransferase), *erm(B)* (rRNA methyltransferase), and *tet(32)*, *tet(O)* and *tet(Q)* (tetracycline resistant ribosomal protection protein). *aad9* (aminoglycoside nucleotidyltransferase), *catA13* (chloramphenicol acyltransferase) and *dfrF* (dihydrofolate reductase) were detected at baseline but not after seven-day vancomycin use. These ARGs positively correlated with bacterial species whose relative abundance decreased under vancomycin use, such as *Blautia sp. SC05B48, [Ruminococcus] gnavus* and *Faecalibacterium prausnitzii*, species that are all susceptible to vancomycin. With the recovery and regrowth of these species during the eight-week supplementation period, these ARGs could be detected again post supplementation in both groups, indicating that these may be carried by the recovered species. The fact that certain ARGs were more detected during vancomycin use whilst others were less detected could be an explanation for the fact that overall, resistome richness and diversity did not change significantly.

Finally, our results showed correlations between ARGs and bacterial species across the entire study: *aadE* (aminoglycoside nucleotidyltransferase) was correlated with *Faecalibacterium prausnitzii*, a finding that corresponds to literature. Bai et al. (2022) show presence of *aadE* and thus implicated aminoglycoside resistance in a pan-genome analysis of *F. prausnitzii* strains.^37^ The correlation between *Roseburia intestinalis* and *tet(O)* (conferring tetracycline resistance) is supported by the literature as well: this gene has recently been identified in the genome of *R. intestinalis.* ^38^ Finally, pan-core genomic analysis of *Ruminococcus gnavus* has previously showed clear *tet(O)* presence in many strains.^38^

Interestingly, limited vancomycin resistance genes were detected in our current analyses, as these genes were detected in only one sample at baseline and two at follow-up. This is in contrast to our previous work in a comparable study population where we were able to detect *vanB* in more than half of the volunteers.^11^ The difference may be explained by the techniques used, as previously, qPCR was used for the detection of *vanB*. Targeted qPCR has been shown to have higher sensitivity compared to the higher coverage of metagenomic sequencing.^39^

In our current study, eight-week 2′-FL supplementation had no detectable effect on resistome richness and diversity. No ARGs were detected that differentially changed between the placebo and the 2′-FL group. The *tet(40)* gene (tetracycline efflux pump) had significantly expanded after eight-week placebo, but this expansion occurred to a lesser extent in the 2′-FL group as well. *tet(O)* on the other hand, was not detected after vancomycin use in the 2′-FL group but could be detected in 8/9 individuals after eight-week supplementation. In the placebo group, detection of *tet(O)* changed from 2 individuals after vancomycin use to 9 post supplementation. The recovery pattern of ARG presence shown in this study is similar to results from Palleja et al. (2018), which showed expansion of glycopeptide ARGs after four-day antibiotic cocktail use in healthy adults and the expansion of genes encoding multidrug efflux pumps (MEPs) four days after treatment cessation.^10^ During the follow-up recovery period, the ARGs returned to baseline levels. Interestingly, ARG-carriage was associated with changes in the odds of survival of specific bacterial species. One important difference with our study is that ARGs were grouped into families working against the three antibiotics used in the study (vancomycin, gentamicin and meropenem), so no information could be gathered about specific ARGs.

Our study is one of the first investigating the impact of prebiotic supplementation on the gut resistome, showing no detected differential effect of eight-week 2′-FL supplementation on gut resistome richness, diversity and composition compared to placebo after antibiotic use. The evidence in the literature is sparse: one study showed a decrease in resistome richness and diversity in children with obesity following a diet including prebiotics and whole-grain products for at least 30 d, and this was linked to a lower abundance of ARG-carriers and expansion of carbohydrate-utilizers such as *Bifidobacterium.* ^14^ However, from the results, it cannot be concluded whether the beneficial effects are due to prebiotic intake or due to the healthy diet in general.

One limitation of this work is the fact that only three sampling points were included in the analyses instead of a total of six, as was done in the original study, which showed transient effects of 2′-FL supplementation during the eight-week supplementation period.^15^ By not including these timepoints, interesting changes occurring during the early recovery period may have been missed. An additional factor to take into account is the effect of diet, and while habitual diet did not change during the study, as reported previously, inter-individual differences in dietary intake could impact the observed changes in the gut microbiome and resistome.^15^

For future work, resistome analyses should be included in studies investigating the gut microbiome, and this should be feasible, as metagenomic sequencing methods have become more commonly used. It is clear that antibiotic use impacts both the gut microbiome and the resistome, and it has been shown previously that ARG load may differ based on disease state.^40^ In order to elucidate their potential role, presence and spread of ARGs across populations should be studied more in-depth. Additionally, bacteria that have become difficult to treat owing to acquired resistance are especially relevant for further investigation.^41^ Insight into the presence and spread of these bacteria and the mechanisms through which they may pass on resistance will be beneficial for the clinical setting.

To conclude, our analyses showed that seven-day vancomycin use clearly disrupted gut microbial composition and diversity. Conversely, no changes were seen in the estimated richness and diversity of the resistome. However, the presence of specific ARGs changed during antibiotic use and subsequent eight-week supplementation, with no detectable difference between 2′-FL and the placebo. Our results further underline the detrimental impact of antibiotic use and corroborate the need to lower the non-essential use of antibiotics. Further research is needed to gain more insight into the presence and spread of resistant bacteria, how these bacteria may affect host health and how they may potentially be treated with alternatives to antibiotics. Whether prebiotics could play a role in this, perhaps by stimulating the growth of non-resistance-carrying species, needs to be investigated in more detail by selecting a broader range of prebiotic compounds.

## Supplementary Material

Supplementary material SupplTable 2.xlsx

Supplementary material SupplFig 1.tif

Supplementary material SupplTable 1.xlsx

Supplementary material SupplFig 3.tif

Supplementary material SupplTable 4.xlsx

Supplementary material SupplTable 3.xlsx

Supplementary material SupplFig 4.tif

Supplementary material SupplFig 2.tif

Supplementary material Supplementary Material

## Data Availability

The whole metagenome shotgun sequencing data for this study have been deposited in the European Nucleotide Archive (ENA) at EMBL-EBI under accession number PRJEB108131. Other data can be made available by the corresponding author upon reasonable request.
