# Supplementary material for: Modulation of the gut microbiome and resistome by antibiotic use, but not by subsequent 2′-fucosyllactose intake, in adults with overweight or obesity
Source: Gut Microbes. 2026 Oct 15;18(1):2746814. doi: 10.1080/19490976.2026.2746814 (PMC13679327; doi:10.1080/19490976.2026.2746814)
Supplement: Supplementary file 1 [file KGMI_A_2746814_SM4946.docx]

**Supplementary material**

Supplementary figures

Supplemental Figure 1: Inter-individual Jaccard Dissimilarity per timepoint on bacterial species level. Sample size n=36 for both the 2’-FL and the placebo group. Comparisons where made within groups over-time. 2’-FL: 2’-fucosyllactose.

Supplemental Figure 2: Relative abundance of bacterial species throughout the study period. Sample size n=9 for both the 2’-FL and the placebo group. 2’-FL: 2’-fucosyllactose.

Supplemental Figure 3: Inter-individual resistome Jaccard dissimilarity per timepoint. Sample size n=36 for both the 2’-FL and the placebo group. 2’-FL: 2’-fucosyllactose.

Supplemental Figure 4: Resistome ARG (total count) throughout the study period. Sample size n=9 for both the 2’-FL and the placebo group. 2’-FL: 2’-fucosyllactose.
